# A 7000‐year history of changing plant trait composition in an Amazonian landscape; the role of humans and climate

**DOI:** 10.1111/ele.13251

**Published:** 2019-03-18

**Authors:** Masha T. van der Sande, William Gosling, Alexander Correa‐Metrio, Jamir Prado‐Junior, Lourens Poorter, Rafael S. Oliveira, Lucas Mazzei, Mark B. Bush

**Affiliations:** ^1^ Institute for Global Ecology Florida Institute of Technology Melbourne FL USA; ^2^ Institute for Biodiversity & Ecosystem Dynamics University of Amsterdam Amsterdam The Netherlands; ^3^ Forest Ecology and Forest Management Group Wageningen University and Research P.O. Box 47 6700 AA Wageningen The Netherlands; ^4^ Instituto de Geología Universidad Nacional Autónoma de México Coyoacán Ciudad de México CP 04510 Mexico; ^5^ Biology Institute Federal University of Uberlandia Uberlandia Brazil; ^6^ Department of Plant Biology Institute of Biology CP 6109 University of Campinas– UNICAMP 13083‐970 Campinas SP Brazil; ^7^ Embrapa Amazônia Oriental Travessa Enéas Pinheiro S/N° 100 Belém CEP 66095 Pará Brazil

**Keywords:** Amazon, charcoal, climate change, erosion, fire activity, fossil pollen, functional traits, human disturbance, Peru, tropical forest

## Abstract

Tropical forests are shifting in species and trait composition, but the main underlying causes remain unclear because of the short temporal scales of most studies. Here, we develop a novel approach by linking functional trait data with 7000 years of forest dynamics from a fossil pollen record of Lake Sauce in the Peruvian Amazon. We evaluate how climate and human disturbances affect community trait composition. We found weak relationships between environmental conditions and traits at the taxon level, but strong effects for community‐mean traits. Overall, community‐mean traits were more responsive to human disturbances than to climate change; human‐induced erosion increased the dominance of dense‐wooded, non‐zoochorous species with compound leaves, and human‐induced fire increased the dominance of tall, zoochorous taxa with large seeds and simple leaves. This information can help to enhance our understanding of forest responses to past environmental changes, and improve predictions of future changes in tropical forest composition.

## Introduction

Tropical forests are shifting in species composition (Laurance *et al*. 2004; Nelson 2005; Feeley *et al*. 2011; van der Sande *et al*. 2016) and carbon sequestration capacity (Brienen *et al*. 2015) probably due to changes in external conditions (e.g. temperature increase and precipitation decrease). Evaluating shifts in the trait values of species can shed light on why and how forests are changing, as these shifts depend on underlying environmental change. For example the relative drought tolerance of species is strongly linked to their distribution along rainfall gradients (Esquivel‐Muelbert *et al*. 2017), community‐mean leaf nutrient concentrations decrease with temperature along elevation (Asner *et al*. 2017; Enquist *et al*. 2017) and community‐mean wood density decreases with soil fertility and water availability (Muller‐Landau 2004; van der Sande *et al*. 2018; Raymundo *et al*. 2019). Based on temporal shifts in trait composition, studies have hypothesised that forests are changing due to increasing drought (Enquist & Enquist 2011; Esquivel‐Muelbert *et al*. 2017), increasing CO_2_ concentrations (Laurance *et al*. 2004), and/or recovery from historic disturbances (van der Sande *et al*. 2016). Such divergences in results may arise because these studies are limited by short timescales (years to decades) that are well below the turnover time of most tropical trees (typically 100–300 years; Hartshorn 1978), and because of strong and often unquantified human influence in current ecosystems. Here, we apply a novel approach to link species abundance data from fossil pollen records spanning millennia with data on environmental change and functional traits, to evaluate long‐term effects of climate and humans on the trait composition of tropical forest landscapes.

Forest responses to external changes are complex and often hard to observe and understand. Long‐term studies based on fossil pollen data (by coring lakes or bogs in which pollen have accumulated and are preserved over time) have traditionally focused on temporal changes in abundance of individual taxa. Such studies show responses of taxa to changes in climate and human influence (e.g. Watling *et al*. 2017; Smith & Mayle 2018). To enhance our functional understanding of forest responses to environmental changes, we can make use of plant traits (Violle *et al*. 2007). If environmental conditions change, we would expect that (1) at the *taxon‐level*, taxa would increase or decrease in abundance depending on their traits and (2) at the *community‐level*, the community‐weighted mean trait would change.

Here, we link palaeoecological data with functional traits from a human‐dominated tropical forest landscape in the lowland Peruvian Amazon, using 7000 years of fossil pollen data from Lake Sauce. We use the abundance of fossil charcoal particles as proxy for fire activity, the width of the annual sediment bands as proxy for erosion rate, and δ^18^O measurements from a nearby cave as proxy for precipitation (van Breukelen *et al*. 2008; Appendix S1). Previous studies of this region demonstrated that the surrounding vegetation remained at least partially forested, but has been exposed to significant droughts (Parsons *et al*. 2018) and heavily influenced by human activity for much of the last 7000 years (Bush *et al*. 2016, 2017). Human influence in the region was evident from anthropogenic fire events, high sedimentation rates due to erosion and the presence of fossil maize pollen.

We address two questions related to the taxon level and the community level. While taxon‐level analyses are mostly used in palaeoecological studies and demonstrate how environment‐trait interactions determine species abundance, community‐level analyses demonstrate how an average tree in the forest (in terms of traits, and weighted by abundance) relates to environmental conditions. First, we ask: how do traits determine taxon‐level responses to climate and human disturbance (erosion rate and fire activity)? We hypothesise that strong drought would cause most severe negative effects on the abundance of taxa with traits related to high‐resource requirements and fast growth (e.g. low wood density, tall adult height, large leaf area) because such ‘acquisitive’ traits are generally associated with low drought tolerance (Table 1). Contrastingly, taxa with traits related to resource conservation (e.g. high wood density, small adult height, small leaves) experience weak or even positive effects of drought because such ‘conservative’ traits are generally associated with high drought tolerance. Human‐induced erosion is probably caused by deforestation and/or forest thinning, which reduce the soil organic content and increase light availability, vapour pressure deficit, the potential for extreme soil moisture deficits and the likelihood of fire. Therefore, harsh environmental conditions due to erosion or fire could increase the abundance of taxa with conservative traits (Table 1). The more open conditions after human‐induced erosion or fire, however, could also enhance the abundance of fast‐growing taxa that can quickly occupy new sites. Second, we ask: how do temporal shifts in community‐mean traits relate to climate and human activity? We expect similar responses as at the taxon level: drought would increase the community‐mean of conservative traits (e.g. high wood density, deciduousness and the proportion with compound leaves) and decrease the community‐mean of acquisitive traits (e.g. leaf area, adult height). Human disturbance could either change community‐mean traits towards being more conservative (e.g. higher wood density) because of harsh conditions, or towards more acquisitive (e.g. high leaf area) due to the availability of open sites (Table 1).

**Table 1:** Hypothesized effects of precipitation (from δ^18^O measurements), erosion rate (from band width) and fire activity (from charcoal abundance) on eight community‐mean traits (wood density, leaf area, adult height, seed mass, leaf compoundness, seed dispersal syndrome, usefulness, and the ratio Poaceae : tree pollen). The hypothesized effects are explained in between brackets

	Precipitation	Erosion rate	Fire activity
Wood density	− (soft‐wooded taxa are more drought vulnerable) (Markesteijn *et al*. 2011; Greenwood *et al*. 2017; Eller *et al*. 2018)	+ (dense‐wooded taxa are more likely to survive the extreme conditions and shallow soils arising from erosion events) or − (soft‐wooded taxa can take advantage of high‐light conditions after disturbance) (Poorter *et al*. 2010)	+ (dense wood is associated with higher fire tolerance and resprouting capacity) (Brando *et al*. 2012)
Leaf area	+ (establishment of fast‐growing taxa with large leaves) (Greenwood *et al*. 2017)	− (increased heath in open areas favors taxa with small leaves that have better convective heath cooling) or + (disturbance favors taxa with large leaves to take advantage of high‐light conditions)	+ (disturbance would favor taxa with large leaves to take advantage of high‐light conditions)
Adult height	+ (less hydraulic limitation allows the establishment of tall taxa with larger conduit sizes) (Bennett *et al*. 2015; Olson *et al*. 2018)	− (erosion may select for small, superficially rooted species)	+ (tall taxa are more likely to escape and survive fire) (Barlow & Peres 2008; Brando *et al*. 2012)
Seed mass	+ (large‐seeded taxa are usually tall and long‐lived and positively related to annual precipitation) (Moles *et al*. 2005)	− (small but many seeds enhance colonization of eroded sites) or + (large seeds are associated with high resprouting capacity) (Westoby 1998; Lahoreau *et al*. 2006)	− (small but many seeds enhance colonization of burned sites) or + (large seeds are associated with high resprouting capacity and fast growth of seedlings, which enhances their chances to escape fire) (Westoby 1998; Lahoreau *et al*. 2006)
Leaf compoundness	− (less need for compound leaves that enhance convective heat cooling and reduce the need for water) (Stowe & Brown 1981)	+ (compound leaves that enhance convective heat cooling provide an advantage in more open landscapes with higher irradiation and temperature) (Stowe & Brown 1981)	+ (compound leaves that enhance convective heat cooling provide an advantage in more open landscapes with higher irradiation and temperature) (Stowe & Brown 1981)
Seed dispersal syndrome	+ Zoochory (wet forests have more animal‐dispersed species) (Howe & Smallwood 1982)	− Zoochory (disturbed and more open forests have less animals, and enhance dispersal distances by wind) (Markl *et al*. 2012)	− Zoochory (disturbed and more open forests have less animals, and enhance dispersal distances by wind) (Markl *et al*. 2012)
Usefulness	No expected effect	+ (useful species would be protected from fire by people)	+ (useful species would be protected from fire by people)
Ratio Poaceae: tree pollen	− (more closed canopy and less Poaceae in the understorey)	+ (more open landscapes would increase Poaceae abundance)	+ (more open landscapes would increase Poaceae abundance)

## Materials and Methods

### Site description

This study is based on data from Lake Sauce in the lowland Peruvian Amazon (6°42′17″S, 76°13′04″W). Lake Sauce lies at 607 m elevation and its main basin is about 2.7 km long by 1.5 km wide, with a maximum depth of 20 m. The climate has a mean annual precipitation of 1475 mm and a dry season from November to December (with on average 180 mm precipitation for the 2 months) (Bush *et al*. 2016). The surrounding vegetation grows on cambisols and is seasonally deciduous tropical forest that has been heavily disturbed by humans throughout most of the last 7000 years.

### Collection of palaeoecological data

In 2003, cores were collected from the deepest part of Lake Sauce. Two parallel cores were collected from the first 10 m depth, and two parallel cores from the next 9.7 m depth, totalling 19.7 m depth. The cores were brought to the laboratory for analyses of fossil pollen, charcoal and band width. Pollen grains and charcoal particles washed or blown in from land are well preserved in sedimentary deposits, and therefore can be used to assess changes in vegetation and fire through time. The age of the cores was reconstructed using ^14^C dating based on macrofossils or bulk samples using Accelerator Mass Spectrometry. The fossil pollen, charcoal and band width data have been published previously by Correa‐Metrio *et al*. (2010) and Bush *et al*. (2016), where a detailed description of collection, laboratory analyses and age reconstruction can be found.

### Fossil pollen data

From the cores, 94 subsamples of 0.5 cm^3^ were taken at every 20 cm of core depth for fossil pollen analysis, covering about 7000 years. For each subsample, a calendar year was determined. The average time interval between subsamples was 72 years (range 12–179 years). Standard protocols (Faegri & Iversen 1989) were used to prepare the pollen samples. Pollen were identified and counted up to 300 grains per subsample. We only focused on taxa occurring in the GlobalTreeSearch of the Botanic Gardens Conservation International (BGCI) and/or occurring as trees in The Plant List. We excluded unidentified taxa, Arecaceae and non‐tree taxa (e.g. Poaceae, shrubs) from the trait analyses because trait data cannot be easily compared between life forms. However, we included Poaceae for calculating the ratio Poaceae : tree pollen (as a measure of landscape openness), and included Arecaceae when calculating the percentage of ‘useful’ genera by using the list produced by Levis *et al*. (2017). Of the total tree pollen, 58% was identified to genus level and 42% to family level.

### Band width – erosion rate

From the core, we analysed the width of sedimentation bands as a measure of sedimentation rate, as it represented erosion from the surrounding landscape. Measurements were made of 8730 bands covering the last 5000 years, during which sediments were strongly laminated (Bush *et al*. 2017). In most years the bands appeared to have been deposited as a couplet, and Bush *et al*. (2017) suggested the bands represented the initiation of the wet season followed by a plankton bloom. This was not a truly varved system (i.e. having annual layers), as some years did not form distinct layers. To match the year of band width data with the year of fossil pollen data, we interpolated band width and calculated values for the same years as we had for the fossil pollen samples, using the *na.approx* function of the *zoo* package in R. We will refer to band width throughout as ‘erosion rate’.

### Charcoal – fire activity

Charcoal abundance was used as measure of fire activity, as high charcoal abundance indicates larger, more frequent, more intense, and/or more severe fires (Whitlock & Larsen 2001). Small charcoal particles (e.g. < 100 μm) provide a regional‐scale indication of fire activity, as these small particles may have been dispersed by wind, whereas larger charcoal particles would mostly wash in, and thus represent local‐scale fire events (Whitlock & Larsen 2001). Here, we used charcoal abundance of particles between 50 and 170 μm, which best matched our regional‐scale fossil pollen signal. Charcoal abundance was determined on subsamples of 0.5 cm^3^ at 470 locations in the soil core, spanning the 7000‐year history. To obtain charcoal abundance data corresponding the years for which we had fossil pollen data, we used two approaches: (1) the closest previous charcoal measurement and (2) the maximum charcoal abundance in the time period preceding the year of the pollen sample. The closest previous charcoal measurement was probably the most relevant if responses to fire were rapid. The maximum value in the time period preceding the pollen measurements, however, may have been most relevant if strong events determine vegetation changes for decades. The two fire activity indices were modestly correlated (*r* = 0.31, *P* = 0.003). We ran all models with both indices and used the model with the lowest AIC and highest *R* ^2^ (Appendix S2). We did not interpolate fire activity to the same years as fossil pollen data because this would have reduced the extreme values and could have underestimated fire effects. We will refer to charcoal abundance throughout as ‘fire activity’.

### δ^18^O data – precipitation

To infer temporal patterns in precipitation we used δ^18^O data published by van Breukelen *et al*. (2008). These δ^18^O data were derived from isotope values in stalagmites in Cueva del Tigre Perdido (5°44′ S, 77°30′ W), 145 km from our site and with similar climate to our site (Appendix S3). High values of δ^18^O indicate drier periods with low precipitation, and low values indicate wetter periods with high precipitation. We used the 383 δ^18^O measurements taken within the timeframe of our fossil pollen (i.e. up to 6860 years before present; BP). These δ^18^O values were interpolated similarly as done for band width, to obtain values at the same time points as for fossil pollen data. To have a proxy of ‘precipitation’, which is easier to interpret, we multiplied the δ^18^O by −1. Note that this proxy for precipitation cannot be directly translated to annual precipitation amounts. We will refer to precipitation, fire activity and erosion rate collectively as ‘environmental variables’.

### Plant traits

To evaluate temporal shifts in functional composition, we used six traits that relate to different plant tissues and likely respond differently to underlying environmental changes (Table 1): (1) wood density, which is part of the wood economics spectrum (Chave *et al*. 2009) and associated to drought‐ and shade‐tolerance; (2) leaf area, which is part of the leaf economics spectrum (Wright *et al*. 2004) and associated with light capture; (3) adult height as a strategy to avoid crown fire, enhance fire tolerance due to its association with bark thickness, enhance light exposure, and linkage with drought vulnerability (Rowland *et al*. 2015); (4) seed mass, which enhances establishment success in the forest understory and in fire‐prone ecosystems, (5) leaf compoundness, which is important for the heat balance of the plant as smaller leaflets increase convective heat cooling and (6) seed dispersal syndrome, as zoochorous taxa may be more abundant in undisturbed forests that have higher animal abundance, and wind‐dispersed taxa may be more abundant in disturbed vegetation (Table 1).

Wood density was obtained from the global wood density database (Zanne *et al*. 2009) and previously collected data (van der Sande *et al*. 2016, 2017a). Leaf area (including all leaflets for species with compound leaves) and adult height were obtained from previously collected data and the Botanical Information and Ecology Network (BIEN) database (http://bien.nceas.ucsb.edu/bien/) using the *BIEN* package in R (Maitner *et al*. 2018). Seed mass was obtained from the Seed Mass Database of Kew Royal Botanical Gardens (http://data.kew.org/sid/), and leaf compoundness and seed dispersal syndrome from online herbaria (http://www.tropicos.org/) and expert knowledge. In all cases, only data collected from the Neotropics were used. Leaf area was ln‐transformed prior to analyses to reduce extreme values.

Studies usually determine traits at species level (Pérez‐Harguindeguy *et al*. 2013). Our fossil pollen data, however, were identified to genus or family level. To evaluate the suitability of our traits for use at genus or family level, we tested for phylogenetic signal in our continuous traits (i.e. wood density, leaf area, adult height and seed mass) with Pagel's lambda, which generally performs well for testing phylogenetic signal (Münkemüller *et al*. 2012) (see Appendix S4 for description of analyses). We found significant phylogenetic signal for all traits (*P* < 0.05, Appendix S4), in agreement with earlier studies (Coelho de Souza *et al*. 2016). Phylogenetic signal was strong for wood density (lambda = 0.91), adult height (0.76) and seed mass (0.97) but weak for leaf area (0.17). This finding indicates that using genus‐level and even family‐level average trait values provide better estimates for species within these genera and families than using random trait values. We also evaluated what part of the variation in species traits was explained by genus and by family. This analysis also showed that genus strongly explained variation among species in wood density (*R* ^2^ = 0.77), seed mass (0.73), adult height (0.51), but less in leaf area (0.33; Appendix S5). To obtain accurate trait estimates, genera and families were only included if we had data for at least three species of that genus or family or, for genera with less than nine species, data for at least 30% of the species in that genus. These trait data were used to calculate community‐weighted mean (CWM) traits.

### Community‐weighted mean traits

We calculated average trait values weighted by taxon abundances for each of the 94 years in our fossil pollen dataset. As a measure of taxon abundance, we used pollen counts of tree taxa identified to genus (70 in total). For compoundness, we calculated the % of pollen belonging to tree taxa with compound leaves, for seed dispersal syndrome, we calculated the % of pollen belonging to animal‐dispersed (i.e. zoochorous) tree taxa, and for usefulness, we calculated the % of pollen belonging to genera listed as ‘useful’ by Levis *et al*. (2017). Pollen identified in the sediment represented some taxa for which trait data were unavailable. Nevertheless, for the identified tree taxa, we had wood density data for on average 65% of the counted pollen, leaf area for 92%, adult height for 69%, seed mass for 78%, leaf compoundness for 99.7%, seed dispersal syndrome for 99.7% and usefulness for 100% of the all pollen per year. Besides these CWM traits, we calculated the ratio of Poaceae pollen to tree pollen as a measure of vegetation openness.

Because taxa differ in pollen production, dispersal and pollination strategy, pollen counts do not provide a direct indication of taxon abundance (Bush 1995). Nevertheless, we assume that relative changes in community‐average properties based on pollen abundance reflect relative changes in community‐average properties of the vegetation for the following four reasons. First, taxon‐level relative changes in pollen abundance along an elevation gradient correspond to taxon‐level relative abundance changes in the field (Urrego *et al*. 2011), and we may expect the same for community‐level changes. Second, spatial variation in pollen composition reflects spatial variation in forest type (Bush 2000). Third, a similar approach has been shown to work for temperate forest (Brussel *et al*. 2018). And fourth, along an elevation gradient in Peru, CWM traits based on abundance from modern pollen vs. CWM traits based on abundance from vegetation plots showed similar direction, although with weaker relationships for pollen‐derived CWM traits (M.T. van der Sande *et al*. unpublished data). In other words, absolute CWM trait values cannot be directly translated to CWM traits in the field, but we assume that the direction of changes over time represent the direction of changes that occurred in the surrounding forest landscape. Given a sufficient sample size and especially where strong environmental gradients exist, long‐term trait trends can be documented.

### Analyses

We evaluated effects of environmental variables (precipitation, fire activity, erosion rate) on the functional trait composition at two levels: taxon and community. All analyses were performed in R 3.5.0. At the taxon level, we assessed how environmental variables and traits interacted to influence variation in taxon‐level pollen abundance across years. First, we tested how environmental variables determined variation in taxon abundance across years. We did so using redundancy analysis on the matrix of taxon abundance × year, with environmental conditions per year as constraining variables, based on 19 genera for which we had data of all traits (using the *rda* function of the *vegan* package). Significance of the constraining variables was tested using an anova with 93 restricted permutations in the time series, to control for temporal autocorrelation. Second, to test for interactions between traits and environmental variables, we used Fourth Corner Analysis, which links a year × environment matrix via a year × taxa abundance matrix to a trait × taxa matrix (using the *traitglm* of the *mvabund* package; Wang *et al*. 2012). An interaction between environmental variables and traits indicates that traits determine the abundance response of taxa to environmental conditions. The significance of the interaction between traits and environmental variables on taxon abundance in the Fourth Corner Analysis was tested using an analysis of deviance based on 99 bootstraps, which compared the Fourth Corner Analysis with a model without interaction between traits and environmental variables (Wang *et al*. 2012), using the *anova* function of the *mvabund* package.

At the community‐level, we evaluated effects of environmental variables on variation in CWM traits across years. First, we evaluated multivariate relationships between environmental variables and CWM traits, using a redundancy analysis on the CWM traits × year matrix, with environmental variables as constraining variables. The significance of the constraining variables was tested using an anova with 93 restricted permutations in the time series. For a principal component analysis, see Appendix S6. Second, we built one generalised least square regression model per community property (i.e. for each community‐weighted mean trait with the three environmental variables as predictors, using the *gls* function of the *nlme* package; Pinheiro & Bates 2016). We included a temporal autocorrelation structure using the corCAR1 function, which is designed to deal with a continuous time covariate to account for temporal autocorrelation. Because the sediments were not consistently laminated between 7000 and 5000 BP, we lacked erosion data for that period. The bands that were visible in this section of the core were similar to those seen at 5000–4500 BP. Rather than setting values to zero or missing, we set band width values to 0.22 mm/y, which is the value of the bandwidth at 5000 BP.

To evaluate if these relationships were different from random, we performed permutation tests by randomly shuffling traits across taxa 1000 times (Zelený & Schaffers 2012), for all except the ratio of Poaceae : tree pollen (because this is no trait). After each permutation, we calculated random CWM trait values per year, and ran the regression models. From this output, we calculated the 95% confidence intervals (CI) of environmental effects on random CWM traits, and compared these with the observed environmental effects on CWM traits (Appendix S7). These permutation tests give a conservative estimate of the significance of the environmental effects (ter Braak *et al*. 2018). Additional analyses of relationships between community‐mean traits and environmental variables for three time periods during which environmental variables showed distinct changes were explored (Appendix S8).

Analyses were performed at two taxonomic resolutions: (1) including only tree taxa identified to genus (70 genera) and (2) including tree taxa identified to both genus and family level (96 taxa). Because results were very similar (Appendix S9), traits are well conserved (Appendices S4 and S5), and genus‐average traits are more informative, hereafter we only show genus‐based results.

## Results

### Taxon‐level results

**Figure 1:**
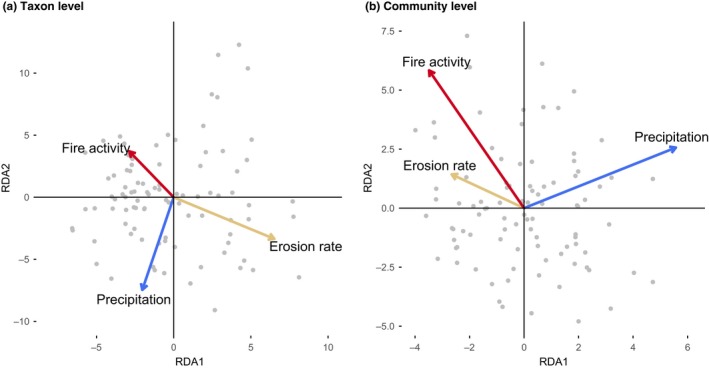
(a) shows results from a redundancy analysis of the effects of precipitation (blue arrow), erosion rate (yellow‐brown arrow) and fire activity (red arrow) on differences among years in pollen abundances of genera. The first axis explains 42% of the variation, the second axis 25%. (b) shows results from a redundancy analysis on the effect of precipitation, erosion rate and fire activity on eight community‐mean trait values (see Table 1). The first axis explained 79% of the variation and the second axis explained 12% of the variation. Each point represents a year (*N* = 94).

None of the environmental variables was significantly related to multivariate variation in taxon composition across years (Fig. 1a, Table 2a). Using Fourth Corner Analysis, we showed that only adult height was significantly positively associated with species abundance at high fire activity (Appendix S10).

**Table 2:** anova results showing the effect of environmental constraining variables on (a) multivariate taxon composition (i.e. pollen abundances of genera across years) and (b) multivariate community‐weighted mean trait composition (i.e. community‐weighted mean values in wood density, adult height, leaf area, seed mass, leaf compoundness, seed dispersal syndrome and usefulness per year). Results were tested using redundancy analyses with the three environmental variables as constraining variables, and significance was obtained using a permuted anova with 93 restricted permutations

Model	Constraining variable	Variance	*F*	*P*
a) Multivariate variation in taxa composition	Precipitation	3.86	1.54	0.734
	Erosion rate	6.55	2.61	0.362
	Fire activity	1.90	0.76	0.840
b) Multivariate variation in community‐weighted mean traits	Precipitation	0.72	5.52	0.170
	Erosion rate	0.39	3.01	0.330
	Fire activity	0.14	1.10	0.468

### Community‐level results

**Figure 2:**
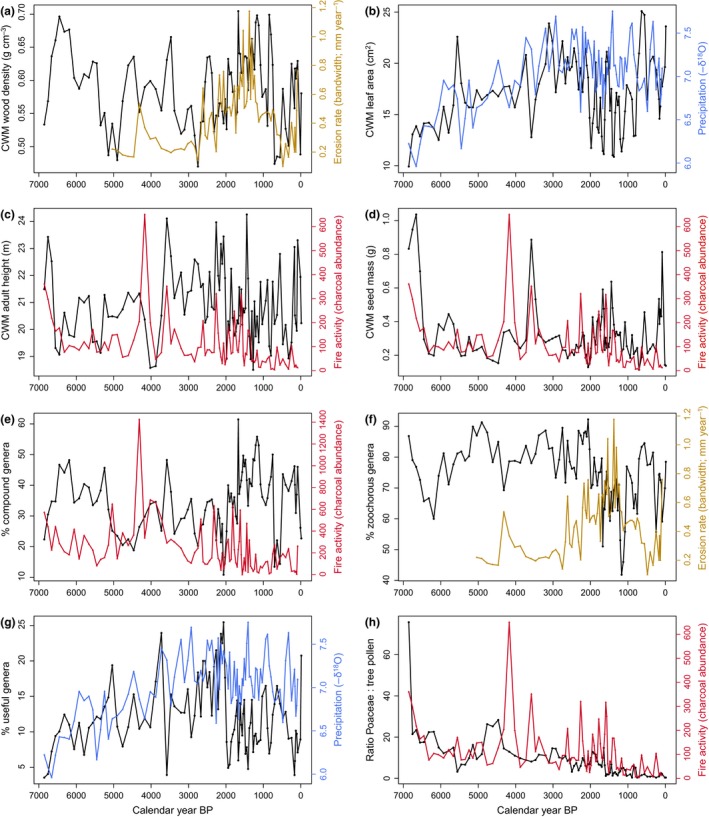
Changes over 7000 years in community‐weighted mean (CWM) (a) wood density, (b) leaf area, (c) adult height and (d) seed mass; changes in the percentage of (e) genera with compound leaves (f) genera that are zoochorous (i.e. animal dispersed) and (g) useful genera; and changes in the ratio of (h) Poaceae to tree pollen (black lines). In each plot, the temporal changes in the best environmental predictor with highest standardised regression coefficient (Appendix S7) is plotted: precipitation (blue lines), erosion rate (yellow‐brown lines) and fire activity (red lines). The missing values of erosion rate for the first 2000 years were replaced by the first measured value (i.e. 0.22) in the regression analyses.

**Figure 3:**
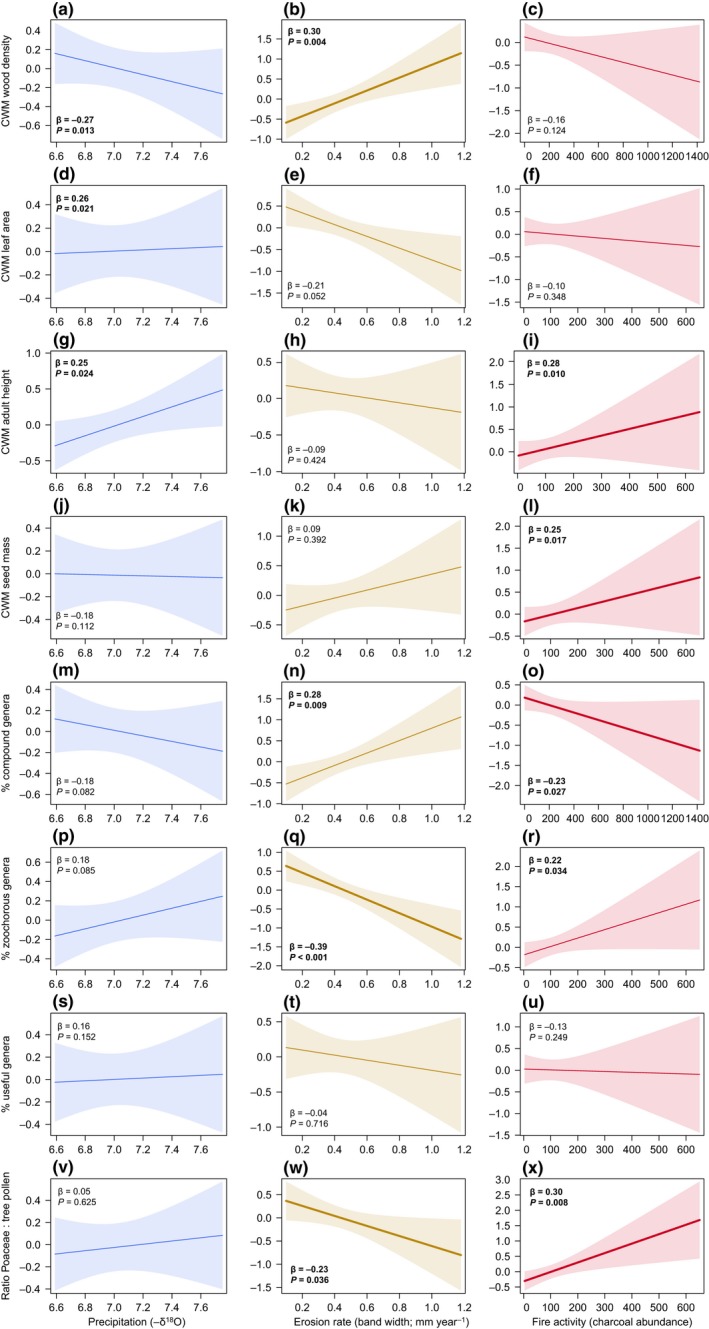
Relationship between environmental variables (blue: precipitation, yellow‐brown: erosion rate, red: fire activity) and community‐weighted mean (CWM) wood density (a‐c), leaf area (d‐f), adult height (g‐i), seed mass (j‐l), % of compound genera (m‐o), % of zoochorous genera (p‐r), % of useful genera (s‐u) and ratio Poaceae : tree pollen (v‐x). The y‐axes show the partial regression residuals (obtained using the *predictorEffect* function of the *effects* package in R), not the absolute values. The standardised regression coefficients (β) and *P*‐values are given per relationship (bold values refer to relationships with *P* ≤ 0.05). Bold regression lines are significant (*P* ≤ 0.05) and fall outside the 95% confidence intervals (Appendix S7). Partial regression slopes correspond to output from the generalised linear models (Appendix S7). For scatter plots of these bivariate relationships, see Appendix S11.

At the community level, we found no effects of environmental variables on multivariate community‐weighted mean (CWM) trait variation across years (Fig. 1b, Table 2b). We then used regression models to evaluate the direction and effect on individual CWM traits (Figs 2 and 3, and Appendix S7 for permutation tests). We found that precipitation was significantly positively related to CWM leaf area and adult height, and negatively to CMW wood density (Fig. 3), although the effect sizes fell just within the 95% confidence intervals of the permutation tests, indicating that these patterns could be generated randomly (Appendix S7). Erosion rate was positively related with CWM wood density and the % of compound‐leaved genera, and negatively with the % zoochorous genera and the ratio of Poaceae : tree pollen (Figs 2 and 3), and only % compound‐leaved genera fell just within the CI. Fire activity was positively related with CWM adult height, seed mass, the % of zoochorous genera and the ratio of Poaceae : tree pollen, and negatively with % of compound‐leaved genera (Figs 2 and 3), and only % zoochorous genera fell just within the CI.

## Discussion

We linked long‐term pollen abundance data with plant functional traits to evaluate how climate and human disturbances determined millennial‐scale shifts in trait composition of a tropical forest landscape. We found weak trait‐environment interaction effects on taxa abundances. The strongest effects, however, were those of human disturbance on community‐level trait composition: high erosion rates led to a more conservative trait composition (e.g. higher community‐weighted mean (CWM) wood density) and fire activity led to a more fire‐avoiding and fire‐tolerant trait composition (e.g. higher CWM adult height).

### Community‐level properties are more sensitive to environmental change than taxon abundance

We expected that both at taxon and community level, traits would explain responses to environmental variables (climate and human disturbance). We found, however, that taxon‐level traits were a weak predictor for climate and human effects on taxa abundance (Appendix S10). Values for the complete set of traits were available for only 19 genera, which may explain the weak relationship between environmental variables, traits and abundance. Moreover, for taxa to be significantly related to environmental variables, a unimodal response to that environmental variable is assumed, as each taxon counts equally in the analysis. Small differences between taxa caused by other factors (e.g. dispersal, pathogens, chance effects) can therefore easily weaken the taxon‐level relationships between environmental variables, traits and abundance.

Multivariate variation in CWM traits was also not predicted by environmental variables. In contrast, individual CWM traits showed strong relationships (β of up to 0.39) with environmental variables (Fig. 3), indicating that CWM trait responses are trait dependent. CWM‐level results may be different from taxon‐level results because CWM traits are less sensitive to unexplained differences between taxa, as more taxa are included and small differences among taxa are more likely to be averaged out. Moreover, for CWM traits to be related to environmental variables, only those taxa with extreme trait values and/or high abundances must respond to environmental variables at each time step. For example the negative relationship between precipitation and CWM wood density can be caused when, with an increase in precipitation, some of the dominant soft‐wooded taxa increase in relative abundance and with drying, some of the dominant hard‐wooded taxa increase in relative abundance. Moreover, the relative abundance of a taxon can increase simply because of reduced abundance in another taxon, which can affect the CWM traits but would not change taxon‐level abundance. Hence, taxon‐level and community‐level analyses explain different aspects of the same story; taxon‐level analyses test for consistent interactions between traits and environmental variables across taxa, whereas community‐level analyses provide a picture of the average forest community, weighted mainly by the dominant taxa. Here, we show that community‐level changes are more sensitive to external changes in climate and human disturbance. These findings indicate that community‐level analyses provide a valuable additional step building on taxon‐level analyses – which are mostly used in palaeoecological studies – to enhance understanding of forest responses to environmental changes.

### Human disturbance has stronger effects on community‐mean traits than climate

We expected that precipitation would favour drought‐vulnerable taxa that are tall, have low wood density, and simple and large leaves. We found, however, that precipitation had no effect on multivariate variation in CWM traits (Fig. 1b), and all significant effects on single CWM traits fell slightly within the conservative confidence intervals of permutation tests (Appendix S7). Nevertheless, precipitation tended to decreased CWM wood density and increase CWM leaf area and adult height (Appendix S7; Fig. 3a,d,g). This indicates that during times of increased precipitation the forest may change from high abundance of slow‐growing, drought‐tolerant taxa towards higher abundance of fast‐growing, drought‐vulnerable taxa. Hence, relationships between community‐mean properties and precipitation that have been observed spatially (ter Steege *et al*. 2006), seem to be similar for temporal responses of forest communities to changes in climate. This is useful for predicting forest responses to future climate change.

We found none of the other expected effects of precipitation, however, indicating that at long temporal scales tropical forests are more resistant to changing climate than is often thought and found at short temporal scales (Nepstad *et al*. 2007; Phillips *et al*. 2010; Malhi *et al*. 2014; Rowland *et al*. 2015). Instead, we found strong effects of human disturbance (through erosion and fire activity) on individual CWM traits.

### Disentangling human effects on community‐mean traits

Erosion rate and fire activity are both indicators of human presence and disturbance. Occasionally large fires were probably used to clear land, and/or were a consequence of extreme droughts (Bush *et al*. 2015). Smaller fires may have been used repeatedly to keep the land or forest understory open and enhance soil fertility. High erosion rates were probably caused by forest thinning and/or land clearance for cultivation or construction of villages (Bush *et al*. 2016).

Interestingly, erosion and fire had different effects on CWM traits (Figs 2 and 3). Fire activity increased CWM adult height, seed mass and % of zoochorous genera, probably because (1) tall and long‐lived taxa have thicker barks and are therefore more likely to survive fire events (Poorter *et al*. 2014; Rosell 2016), (2) large seeds have higher survival probability in fire events (Ribeiro *et al*. 2015) and have enhanced resprouting ability after fire events (Lahoreau *et al*. 2006) and (3) zoochorous genera have larger seeds (Westoby *et al*. 1996) and may therefore have high seed survival and resprouting ability.

We find that erosion does not affect CWM adult height and seed mass, but enhances CWM wood density and the % compound‐leaved genera, while reducing the % zoochorous genera (Figs 2 and 3). Erosion can decrease soil organic matter and fertility, and due to the presence of more open areas, erosion is associated with vapour pressure deficit and, hence, drought stress. These changes may explain the increase in abundance of taxa that are drought tolerant (high wood density), have efficient convective heath cooling (compound leaves), and are wind or auto dispersed. Erosion rate and fire activity are uncorrelated (*r* = −0.03, *t* = −0.30, *P* = 0.76). This decoupling of erosion and fire is also supported by their effects on the ratio of Poaceae : tree pollen. Poaceae : tree pollen responds positively to fire activity, indicating that fire opens up the vegetation (Figs 2h and 3x), but responds negatively to erosion rate (Fig. 3w), possibly because of intentional weeding and/or use of agroforestry systems that suppress weeds (Levis *et al*. 2018). Alternatively, humans prefer living in more open areas, explaining the positive relationship between Poaceae abundance and fire activity.

In sum, human disturbance through erosion positively relates with abundance of conservative taxa with high wood density, compound leaves, and that are wind or auto dispersed because of more shallow soils and drought stress. Human disturbance through fire, however, mainly increases abundance of fire‐tolerant taxa with large seeds that can survive fire, and increases abundance of tall taxa that are more fire‐tolerant and can avoid crown damage. Since the functional composition of tropical forests is strongly linked with functions they provide (e.g. carbon sequestration; Poorter *et al*. 2017; van der Sande *et al*. 2017b), human disturbance likely has strong consequences for long‐term forest functioning.

### Future perspectives

We developed an approach to evaluate long‐term shifts in forest composition by linking fossil pollen data with functional traits. This approach could enhance our understanding of the functional response of ecosystems to natural and anthropogenic environmental changes, and develop more mechanistic predictions of forest change. To date, the approach is limited by the taxonomic resolution of the pollen and the availability of trait data. Nevertheless, trait data are becoming more readily available, and pollen are identified with increasing taxonomic resolution. These advances will pave the way for more detailed analyses of millennial‐scale changes in trait composition in relation to climate and human disturbance. Here, we show that increased precipitation tends to favour the relative abundance of drought‐vulnerable taxa, that human‐induced erosion increases the relative abundance of conservative taxa, and that human‐induced fire enhances the relative abundance of taxa that can survive and/or escape fire. Effects of human disturbance on CWM traits were stronger than precipitation, indicating that management decisions to reduce and regulate human influence will be important to protect tropical forest ecosystems. This information can help us not only understand forest responses to past environmental changes, but also improve predictions of future changes in tropical forest composition.

## Authorship

MvdS, WG and MBB conceived the idea of the study; ACM and MBB collected and processed the fossil pollen, charcoal and band width data, MvdS, LM, LP and JPP contributed trait data, MvdS analysed the data and wrote the manuscript; all authors discussed the results and contributed substantially to the revisions.

## Data Availability

All data used in the analyses are published in the DANS repository established by the Dutch Royal Academy of Sciences: https://doi.org/10.17026/dans-z3z-n3k2.
