# Supplementary material for: A 7000‐year history of changing plant trait composition in an Amazonian landscape; the role of humans and climate
Source: Ecol Lett. 2019 Mar 18;22(6):925–35. doi: 10.1111/ele.13251 (PMC6850629; doi:10.1111/ele.13251)
Supplement: Supplementary file 1 [file ELE-22-925-s001.docx]

**Supporting Information Appendices.**

*M.T. van der Sande, W. Gosling, A. Correa-Metrio, J. Prado-Junior, L. Poorter, R.S. Oliveira, L. Mazzei, and M.B. Bush. 2019. A 7000-year history of changing plant trait composition in an Amazonian landscape; the role of humans and climate. Ecology Letters.*

**Appendix S1:** Temporal changes in precipitation (derived from d18O multiplied by -1; van Breukelen *et al.* 2008), erosion rate (derived from band width) and fire activity (from charcoal abundance) over the last 7000 years. Erosion rate and fire activity are derived from the sediment core of lake Sauce, and precipitation is derived from measurements 145 km from lake Sauce.


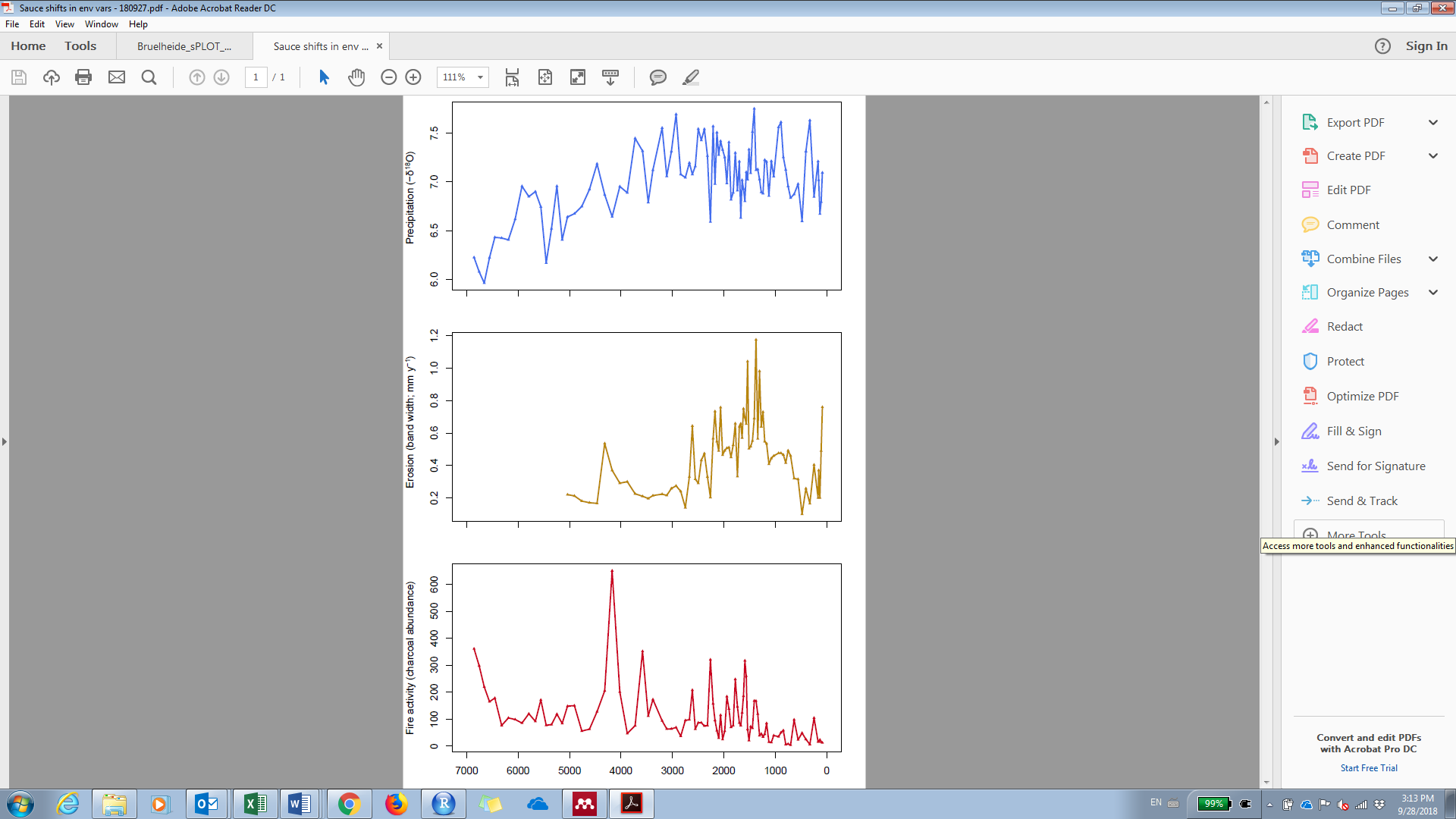


**Appendix S2:** Fit of generalized least square regression analyses for different community-weighted mean (CWM) traits, with two fire activity indices (‘previous’=closest previous fire event, ‘maximum’=maximum fire event in preceding period; see Methods for more info). Per model, the Akaike Information Criterion (AIC) and explained variation (R^2^) are given. The best model per trait is highlighted in bold and presented in the manuscript.

| **Response variable** | **Fire activity index** | **R^2^** | **AIC** |
| --- | --- | --- | --- |
| CWM wood density | previous | 0.11 | 277.55 |
|  | **maximum** | **0.14** | **275.14** |
| CWM leaf area | **previous** | **0.10** | **278.76** |
|  | maximum | 0.10 | 279.26 |
| CWM adult height | **previous** | **0.09** | **281.31** |
|  | maximum | 0.04 | 284.68 |
| CWM seed mass | **previous** | **0.12** | **276.98** |
|  | maximum | 0.08 | 281.07 |
| % compound-leaved genera | previous | 0.11 | 277.95 |
|  | **maximum** | **0.13** | **275.50** |
| % zoochorous genera | **previous** | **0.17** | **271.29** |
|  | maximum | 0.14 | 274.69 |
| Ratio Poaceae : tree pollen | previous | 0.24 | 263.10 |
|  | **maximum** | **0.27** | **260.13** |
| % useful genera | **previous** | **0.05** | **283.53** |
|  | maximum | 0.04 | 284.72 |

**Appendix S3:** Differences in annual rainfall distribution between Lake Sauce (black line) and the cave El Tigre Perdido (red line), from where δ^18^O data were obtained (van Breukelen *et al.* 2008). Monthly rainfall data were obtained from WorldClim (averaged over 1970 – 2000). Total annual rainfall at Lake Sauce is 1405 mm, and at El Tigre Perdido 1420 mm.**
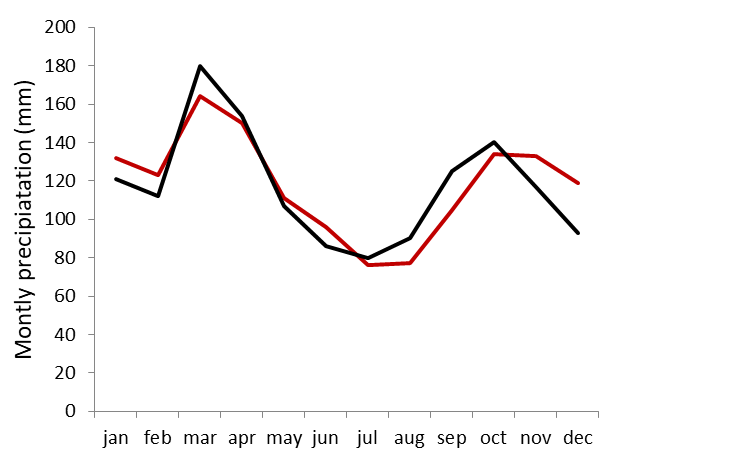
**

**Appendix S4:** Phylogenetic signals in traits (wood density, leaf area, adult height, and seed mass). To test for phylogenetic signals, we used all available trait data from Neotropical genera and families to increase the sample size for phylogenetic analyses and the generality of the results. A phylogenetic tree was constructed based on the phylogeny from Zanne *et al.* (2014), using the *congeneric.merge* of the *pez* package (Pearse *et al.* 2015) in R to construct a tree based on the species in our trait dataset. For wood density, we had 2245 species from 620 genera, for leaf area we had 1032 species from 248 genera, for adult height we had 384 species from 58 genera, and for seed mass we had 1120 species from 65 genera. We tested for phylogenetic signal per trait using the *phylosig* function of the *phytools* package in R. Lambda varies between 0 and 1, with low values indicating low phylogenetic signal in the trait data given the original phylogenetic tree, and high values indicating relatively more phylogenetic signal in the trait data given the original tree.

| **Trait** | **Pagel's lambda** | **P-value** |
| --- | --- | --- |
| Wood density | 0.91 | <0.001 |
| Leaf area | 0.17 | <0.001 |
| Adult height | 0.76 | <0.001 |
| Seed mass | 0.97 | <0.001 |

**Appendix S5:** Explanatory power (R^2^) of Genus and Family on variation in continuous traits among species. The same data were used as in Appendix S4, and tested using 2 linear regression models per trait, with either Genus or Family as predictor variable.

| **Response variable** | **Predictor variable** | **R^2^** |
| --- | --- | --- |
| Wood density | Genus | 0.77 |
|  | Family | 0.36 |
| Leaf area | Genus | 0.33 |
|  | Family | 0.21 |
| Adult height | Genus | 0.51 |
|  | Family | 0.43 |
| Seed mass | Genus | 0.73 |
|  | Family | 0.60 |

**Appendix S6:** Visualization of a principal component analysis including all community-average traits and environmental variables (precipitation, erosion rate, fire activity).
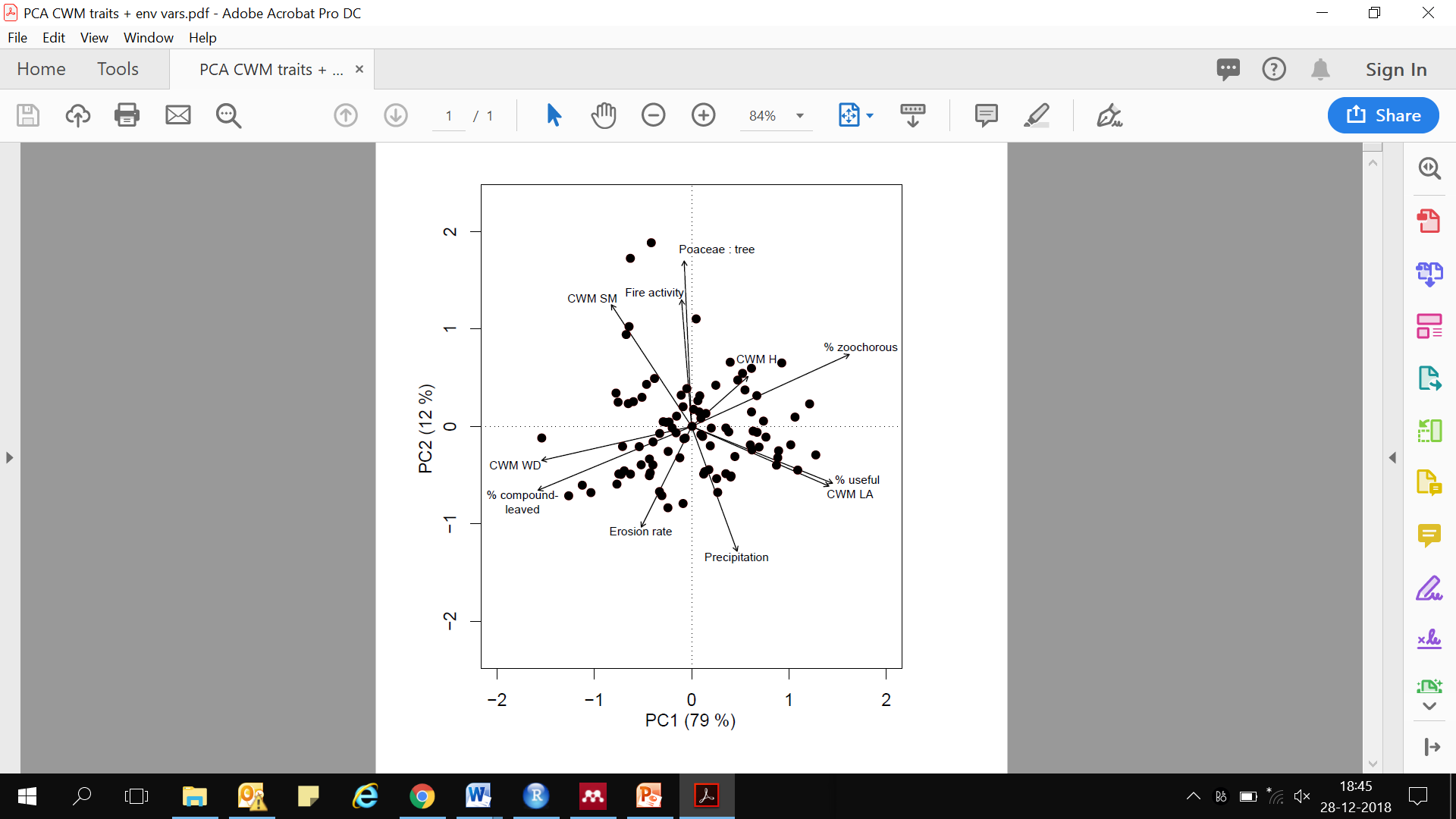


**Appendix S7:** Results from generalized least squares regression analyses of the ‘best’ models (see Appendix S2), including the standardized regression coefficient (Std. coeff), standard error (SE), t-value and P-value. The results correspond with Fig. 3 in the main text. Per model, temporal changes in the predictor variable with strongest standardized coefficient and the response variable are visualized in Fig. 2, and partial regression relationships in Fig. 3.

Modified permutation tests, as proposed by Zelený & Schaffers (2012) to correct for inflated Type I error in CWM trait ~ environment relationships, were performed per model. Here, the 95% confidence interval (CI) of the distribution of regression coefficients from permutation tests are given. Observed standardized coefficients that fall outside the 95% CI are significantly different from random.

|  |  | **Observed** |  |  |  | **Permutation tests** | |
| --- | --- | --- | --- | --- | --- | --- | --- |
| **Response variable** | **Predictor variable** | **Std. coeff** | **SE** | **t-value** | **P-value** | **2.5% CI** | **97.5% CI** |
| CWM wood density | Precipitation | -0.265 | 0.10 | -2.54 | **0.013** | -0.281 | 0.290 |
|  | **Erosion rate** | **0.304** | **0.10** | **2.95** | **0.004** | **-0.257** | **0.240** |
|  | Fire activity | -0.157 | 0.10 | -1.55 | 0.124 | -0.213 | 0.209 |
| CWM leaf area | Precipitation | 0.258 | 0.11 | 2.34 | **0.021** | -0.279 | 0.287 |
|  | Erosion rate | -0.207 | 0.11 | -1.97 | 0.052 | -0.289 | 0.283 |
|  | Fire activity | -0.099 | 0.10 | -0.94 | 0.348 | -0.251 | 0.217 |
| CWM adult height | Precipitation | 0.256 | 0.11 | 2.30 | **0.024** | -0.277 | 0.285 |
|  | Erosion rate | -0.085 | 0.11 | -0.80 | 0.424 | -0.291 | 0.276 |
|  | **Fire activity** | **0.281** | **0.11** | **2.65** | **0.010** | **-0.231** | **0.234** |
| CWM seed mass | Precipitation | -0.175 | 0.11 | -1.61 | 0.112 | -0.283 | 0.273 |
|  | Erosion rate | 0.089 | 0.10 | 0.86 | 0.392 | -0.251 | 0.275 |
|  | **Fire activity** | **0.253** | **0.10** | **2.44** | **0.017** | **-0.203** | **0.250** |
| % compound genera | Precipitation | -0.184 | 0.10 | -1.76 | 0.082 | -0.304 | 0.284 |
|  | **Erosion rate** | 0.275 | 0.10 | 2.66 | **0.009** | -0.276 | 0.291 |
|  | **Fire activity** | **-0.227** | **0.10** | **-2.24** | **0.027** | **-0.212** | **0.271** |
| % zoochorous genera | Precipitation | 0.184 | 0.11 | 1.74 | 0.085 | -0.305 | 0.298 |
|  | **Erosion rate** | **-0.392** | **0.10** | **-3.88** | **<0.001** | **-0.291** | **0.297** |
|  | Fire activity | 0.217 | 0.10 | 2.16 | **0.034** | -0.251 | 0.222 |
| % useful genera | Precipitation | 0.163 | 0.11 | 1.44 | 0.152 | -0.286 | 0.255 |
|  | Erosion rate | -0.039 | 0.11 | -0.37 | 0.716 | -0.273 | 0.271 |
|  | Fire activity | -0.125 | 0.11 | -1.16 | 0.249 | -0.203 | 0.227 |
| Ratio Poaceae : tree pollen | Precipitation | 0.054 | 0.11 | 0.49 | 0.625 |  |  |
|  | **Erosion rate** | -0.233 | 0.11 | -2.14 | **0.036** |  |  |
|  | **Fire activity** | 0.300 | 0.11 | 2.71 | **0.008** |  |  |

**Appendix S8: Analyses per time period**

To identify time periods that differed in the importance of different environmental variables, we computed breakpoints in the relationship between each environmental variable and time, and based on that we divided the whole time period into four shorter periods: from 7000 – 4465 y BP, from 4465 – 2555 y BP, from 2555 – 1205 y BP, and later than 1205 y BP (Figure S3). We computed breakpoints using the *breakpoints* function from the *strucchange* package(Zeileis *et al.* 2015). For each time period, we ran a generalized least square regression model per community property, as described in the main text.

When dividing the timeframe into 4 distinctive periods, only a few of the significant effects remain (Table S8.1). Precipitation only decreased % of compound genera in the third period from 2555–1205 y BP. Erosion rate decreased % zoochorous genera in the fourth period after 1205 y BP and increased % of compound genera and decreased the ratio Poaceae : tree pollen in the first period from 7000–4465 y. Fire activity increased CWM adult height, CWM seed mass and ratio Poaceae : tree pollen, and decreased % useful genera in the first period. These results indicate that shorter timescales and/or lower sample sizes reduce the ability to detect significant relationships.

| 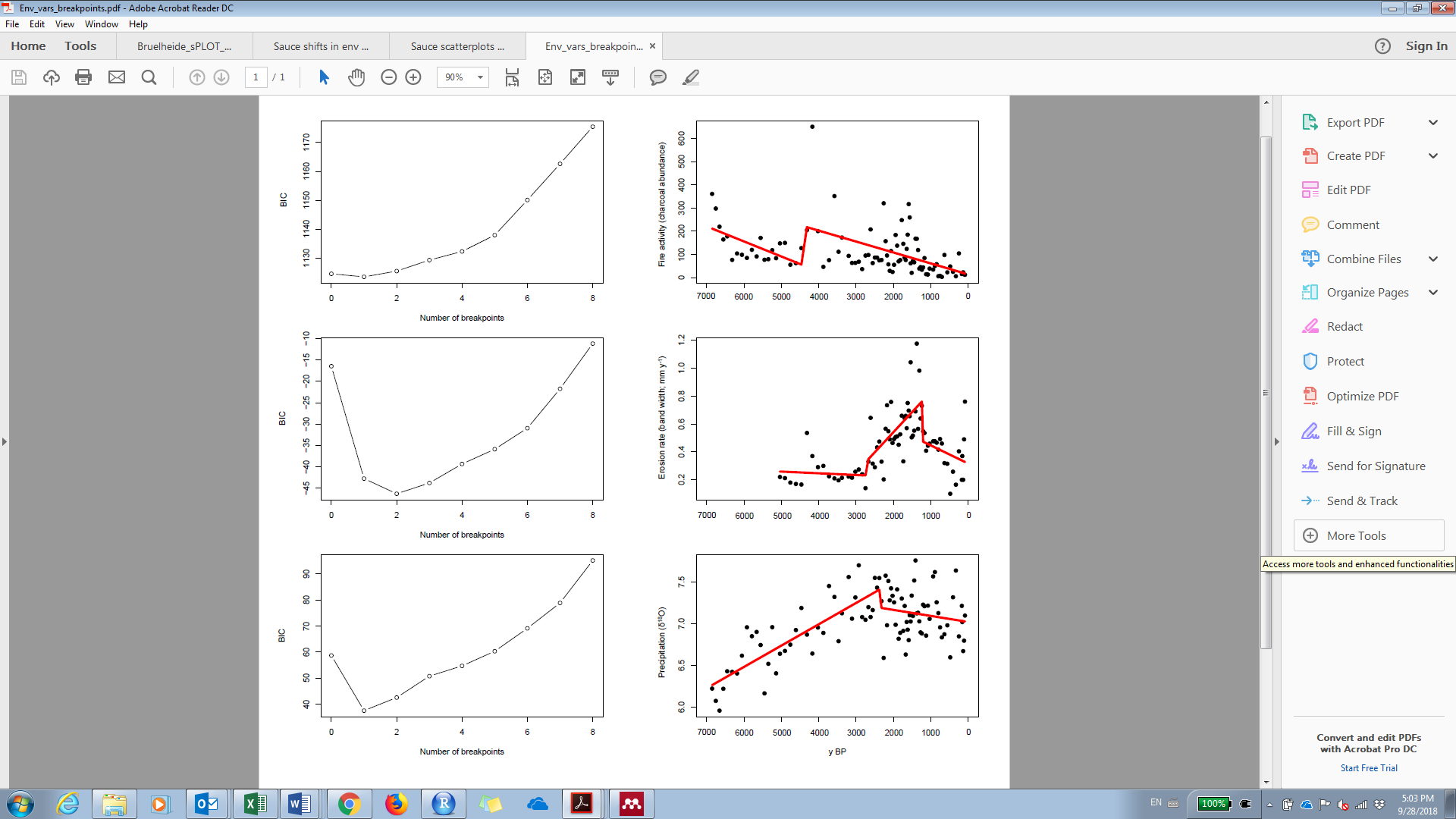 |
| --- |

**Figure S8.1:** Breakpoints in time series of environmental variables (upper row: fire activity, middle row: erosion rate, last row: precipitation). The first column shows changes in Bayesian Information Criterion (BIC) with increasing number of breakpoints. The second column shows the predicted regression relationship based on the most optimal number of breakpoints (i.e. with the lowest BIC). The optimal models for fire activity and precipitation had 1 breakpoint, and for erosion rate 2 breakpoints. Based on these breakpoints, we split the total time series at three points: 4465 y BP (the breakpoint of fire activity), 2555 y BP (the average between the first breakpoints of erosion rate and precipitation), and 1205 y BP (the second breakpoint of erosion rate).

**Table S8.1:** Results from various generalized least regression analyses to evaluate the effect of environmental predictors (precipitation, erosion rate and fire activity) on community-mean traits (wood density, leaf area, adult height, and seed mass for four time periods: 7000 – 4465 y BP, from 4465 – 2555 y BP, from 2555 – 1205 y BP, and 1205-0 y BP. See Appendix S2 for how the time series was divided into these 4 periods.

| **Response variable** | **Model** | **Predictor variable** | **Std. coeff** | **SE** | **t-value** | **P-value** | **R^2^** |
| --- | --- | --- | --- | --- | --- | --- | --- |
| Community-mean wood density | 7000-4465 y BP | Intercept | 0.00 | 0.23 | 0.00 | 1.000 | 0.09 |
|  |  | Precipitation | -0.33 | 0.29 | -1.11 | 0.282 |  |
|  |  | Erosion rate | 0.01 | 0.26 | 0.03 | 0.974 |  |
|  |  | Fire activity | -0.28 | 0.29 | -0.97 | 0.347 |  |
|  | 4465-2555 y BP | Intercept | 0.00 | 0.25 | 0.00 | 1.000 | 0.17 |
|  |  | Precipitation | -0.17 | 0.30 | -0.58 | 0.571 |  |
|  |  | Erosion rate | -0.16 | 0.28 | -0.58 | 0.571 |  |
|  |  | Fire activity | 0.33 | 0.30 | 1.11 | 0.288 |  |
|  | 2555-1205 y BP | Intercept | 0.00 | 0.16 | 0.00 | 1.000 | 0.16 |
|  |  | Precipitation | -0.26 | 0.17 | -1.48 | 0.148 |  |
|  |  | Erosion rate | 0.27 | 0.17 | 1.63 | 0.113 |  |
|  |  | Fire activity | -0.16 | 0.17 | -0.92 | 0.367 |  |
|  | 1205-0 y BP | Intercept | 0.00 | 0.21 | 0.00 | 1.000 | 0.19 |
|  |  | Precipitation | -0.17 | 0.22 | -0.76 | 0.457 |  |
|  |  | Erosion rate | 0.43 | 0.22 | 1.93 | 0.070 |  |
|  |  | Fire activity | 0.07 | 0.22 | 0.31 | 0.762 |  |
| Community-mean leaf area | 7000-4465 y BP | Intercept | 0.00 | 0.21 | 0.00 | 1.000 | 0.25 |
|  |  | Precipitation | 0.22 | 0.26 | 0.82 | 0.423 |  |
|  |  | Erosion rate | -0.08 | 0.23 | -0.32 | 0.752 |  |
|  |  | Fire activity | -0.31 | 0.26 | -1.18 | 0.255 |  |
|  | 4465-2555 y BP | Intercept | 0.00 | 0.25 | 0.00 | 1.000 | 0.19 |
|  |  | Precipitation | -0.09 | 0.29 | -0.29 | 0.775 |  |
|  |  | Erosion rate | 0.08 | 0.28 | 0.29 | 0.778 |  |
|  |  | Fire activity | -0.48 | 0.30 | -1.63 | 0.130 |  |
|  | 2555-1205 y BP | Intercept | 0.00 | 0.17 | 0.00 | 1.000 | 0.06 |
|  |  | Precipitation | 0.06 | 0.18 | 0.33 | 0.744 |  |
|  |  | Erosion rate | -0.22 | 0.18 | -1.25 | 0.222 |  |
|  |  | Fire activity | -0.02 | 0.18 | -0.10 | 0.921 |  |
|  | 1205-0 y BP | Intercept | 0.00 | 0.23 | 0.00 | 1.000 | 0.06 |
|  |  | Precipitation | -0.06 | 0.24 | -0.26 | 0.795 |  |
|  |  | Erosion rate | -0.21 | 0.24 | -0.87 | 0.398 |  |
|  |  | Fire activity | 0.07 | 0.24 | 0.31 | 0.764 |  |
| Community-mean adult height | 7000-4465 y BP | Intercept | 0.00 | 0.21 | 0.00 | 1.000 | 0.28 |
|  |  | Precipitation | 0.12 | 0.26 | 0.45 | 0.661 |  |
|  |  | Erosion rate | -0.29 | 0.23 | -1.26 | 0.225 |  |
|  |  | Fire activity | 0.59 | 0.26 | 2.31 | 0.035 |  |
|  | 4465-2555 y BP | Intercept | 0.00 | 0.26 | 0.00 | 1.000 | 0.12 |
|  |  | Precipitation | 0.33 | 0.31 | 1.08 | 0.301 |  |
|  |  | Erosion rate | -0.14 | 0.29 | -0.50 | 0.625 |  |
|  |  | Fire activity | 0.20 | 0.31 | 0.65 | 0.530 |  |
|  | 2555-1205 y BP | Intercept | 0.00 | 0.17 | 0.00 | 1.000 | 0.09 |
|  |  | Precipitation | 0.21 | 0.18 | 1.17 | 0.252 |  |
|  |  | Erosion rate | -0.18 | 0.18 | -1.03 | 0.309 |  |
|  |  | Fire activity | 0.11 | 0.18 | 0.59 | 0.557 |  |
|  | 1205-0 y BP | Intercept | 0.00 | 0.22 | 0.00 | 1.000 | 0.15 |
|  |  | Precipitation | 0.10 | 0.23 | 0.44 | 0.666 |  |
|  |  | Erosion rate | 0.31 | 0.23 | 1.36 | 0.191 |  |
|  |  | Fire activity | -0.17 | 0.22 | -0.76 | 0.457 |  |
| Community-mean seed mass | 7000-4465 y BP | Intercept | 0.00 | 0.14 | 0.00 | 1.000 | 0.68 |
|  |  | Precipitation | -0.24 | 0.17 | -1.38 | 0.187 |  |
|  |  | Erosion rate | -0.02 | 0.15 | -0.10 | 0.921 |  |
|  |  | Fire activity | 0.67 | 0.17 | 3.88 | 0.001 |  |
|  | 4465-2555 y BP | Intercept | 0.00 | 0.23 | 0.00 | 1.000 | 0.31 |
|  |  | Precipitation | 0.15 | 0.27 | 0.57 | 0.579 |  |
|  |  | Erosion rate | -0.40 | 0.25 | -1.58 | 0.140 |  |
|  |  | Fire activity | 0.54 | 0.27 | 2.00 | 0.069 |  |
|  | 2555-1205 y BP | Intercept | 0.00 | 0.17 | 0.00 | 1.000 | 0.06 |
|  |  | Precipitation | -0.10 | 0.18 | -0.54 | 0.590 |  |
|  |  | Erosion rate | 0.18 | 0.18 | 0.98 | 0.333 |  |
|  |  | Fire activity | 0.09 | 0.18 | 0.47 | 0.644 |  |
|  | 1205-0 y BP | Intercept | 0.00 | 0.21 | 0.00 | 1.000 | 0.18 |
|  |  | Precipitation | -0.10 | 0.22 | -0.46 | 0.652 |  |
|  |  | Erosion rate | 0.26 | 0.22 | 1.15 | 0.265 |  |
|  |  | Fire activity | -0.35 | 0.22 | -1.57 | 0.134 |  |
| Taxon richness | 7000-4465 y BP | Intercept | 0.00 | 0.28 | 0.00 | 1.000 | 0.03 |
|  |  | Precipitation | 0.06 | 0.31 | 0.20 | 0.847 |  |
|  |  | Erosion rate | 0.08 | 0.34 | 0.23 | 0.820 |  |
|  |  | Fire activity | 0.11 | 0.32 | 0.34 | 0.737 |  |
|  | 4465-2555 y BP | Intercept | 0.00 | 0.27 | 0.00 | 1.000 | 0.04 |
|  |  | Precipitation | -0.16 | 0.32 | -0.49 | 0.633 |  |
|  |  | Erosion rate | -0.02 | 0.30 | -0.05 | 0.958 |  |
|  |  | Fire activity | -0.21 | 0.32 | -0.65 | 0.526 |  |
|  | 2555-1205 y BP | Intercept | 0.00 | 0.17 | 0.00 | 1.000 | 0.10 |
|  |  | Precipitation | 0.15 | 0.18 | 0.85 | 0.403 |  |
|  |  | Erosion rate | 0.27 | 0.17 | 1.57 | 0.127 |  |
|  |  | Fire activity | -0.05 | 0.18 | -0.29 | 0.777 |  |
|  | 1205-0 y BP | Intercept | 0.00 | 0.23 | 0.00 | 1.000 | 0.04 |
|  |  | Precipitation | 0.17 | 0.24 | 0.72 | 0.479 |  |
|  |  | Erosion rate | -0.01 | 0.24 | -0.02 | 0.980 |  |
|  |  | Fire activity | -0.09 | 0.24 | -0.39 | 0.701 |  |

**Appendix S9:** Results from generalized least regression analyses to evaluate the effect of environmental predictors (precipitation, erosion rate and fire activity) on community-mean traits (wood density, leaf area, adult height, and seed mass) based on genus-level CWM trait values and genus and family-level CWM trait values. Standardized regression coefficient, standard error (SE), t-value and P-value are given.

| **Response variable** | **Model description** | **Predictor variable** | **Std. value** | **SE** | **t-value** | **P-value** |
| --- | --- | --- | --- | --- | --- | --- |
| Community-mean wood density | Default (genera) | Intercept | 0.00 | 0.10 | 0.00 | 1.000 |
|  |  | Precipitation | -0.24 | 0.10 | -2.27 | **0.026** |
|  |  | Erosion rate | 0.32 | 0.10 | 3.06 | **0.003** |
|  |  | Fire activity | -0.01 | 0.10 | -0.08 | 0.934 |
|  | Genera and families | Intercept | 0.00 | 0.10 | 0.00 | 1.000 |
|  |  | Precipitation | -0.32 | 0.10 | -3.13 | **0.002** |
|  |  | Erosion rate | 0.34 | 0.10 | 3.29 | **0.001** |
|  |  | Fire activity | -0.04 | 0.10 | -0.36 | 0.722 |
| Community-mean leaf area | Default (genera) | Intercept | 0.00 | 0.10 | 0.00 | 1.000 |
|  |  | Precipitation | 0.26 | 0.11 | 2.34 | **0.021** |
|  |  | Erosion rate | -0.21 | 0.11 | -1.97 | 0.052 |
|  |  | Fire activity | -0.10 | 0.10 | -0.94 | 0.348 |
|  | Genera and families | Intercept | 0.00 | 0.10 | 0.00 | 1.000 |
|  |  | Precipitation | 0.27 | 0.11 | 2.41 | **0.018** |
|  |  | Erosion rate | -0.14 | 0.11 | -1.31 | 0.194 |
|  |  | Fire activity | -0.07 | 0.11 | -0.62 | 0.540 |
| Community-mean adult height | Default (genera) | Intercept | 0.00 | 0.10 | 0.00 | 1.000 |
|  |  | Precipitation | 0.27 | 0.11 | 2.38 | **0.019** |
|  |  | Erosion rate | -0.07 | 0.11 | -0.68 | 0.497 |
|  |  | Fire activity | 0.21 | 0.11 | 1.99 | **0.050** |
|  | Genera and families | Intercept | 0.00 | 0.10 | 0.00 | 1.000 |
|  |  | Precipitation | 0.24 | 0.11 | 2.14 | **0.035** |
|  |  | Erosion rate | -0.17 | 0.11 | -1.63 | 0.106 |
|  |  | Fire activity | 0.26 | 0.11 | 2.48 | **0.015** |
| Community-mean seed mass | Default (genera) | Intercept | 0.00 | 0.10 | 0.00 | 1.000 |
|  |  | Precipitation | -0.17 | 0.11 | -1.61 | 0.112 |
|  |  | Erosion rate | 0.09 | 0.10 | 0.86 | 0.394 |
|  |  | Fire activity | 0.25 | 0.10 | 2.45 | **0.016** |
|  | Genera and families | Intercept | 0.00 | 0.10 | 0.00 | 1.000 |
|  |  | Precipitation | -0.13 | 0.11 | -1.25 | 0.215 |
|  |  | Erosion rate | 0.03 | 0.10 | 0.27 | 0.791 |
|  |  | Fire activity | 0.30 | 0.10 | 2.93 | **0.004** |

**Appendix S10:** Significance of interaction effects between taxon traits (wood density, adult height, leaf area, seed mass, compound-leaved, zoochoric, and usefulness) and the response of taxa abundance to precipitation, erosion rate, and fire activity. Results were derived from Fourth Corner Analysis. P-values and Holm-corrected P-values are given.

| **Trait** | **Environment** | **P-value** | **P-value adjusted** |
| --- | --- | --- | --- |
| Wood density | Precipitation | 0.054 | 0.915 |
|  | Erosion rate | 0.533 | 1.000 |
|  | Fire activity | 0.215 | 1.000 |
| Leaf area | Precipitation | 0.830 | 1.000 |
|  | Erosion rate | 0.060 | 1.000 |
|  | Fire activity | 0.360 | 1.000 |
| Adult height | Precipitation | 0.726 | 1.000 |
|  | Erosion rate | 0.128 | 1.000 |
|  | Fire activity | **0.018** | 0.356 |
| Seed mass | Precipitation | 0.320 | 1.000 |
|  | Erosion rate | 0.435 | 1.000 |
|  | Fire activity | 0.460 | 1.000 |
| Compound-leaved | Precipitation | 0.997 | 1.000 |
|  | Erosion rate | 0.204 | 1.000 |
|  | Fire activity | 0.856 | 1.000 |
| Zoochoric | Precipitation | 0.956 | 1.000 |
|  | Erosion rate | 0.880 | 1.000 |
|  | Fire activity | 0.673 | 1.000 |
| Usefulness | Precipitation | 0.654 | 1.000 |
|  | Erosion rate | 0.630 | 1.000 |
|  | Fire activity | 0.939 | 1.000 |

| **Appendix S11:** Scatterplots between precipitation (blue), erosion rate (brown-yellow), fire activity (red) and each of the response variables shown in Fig. 3.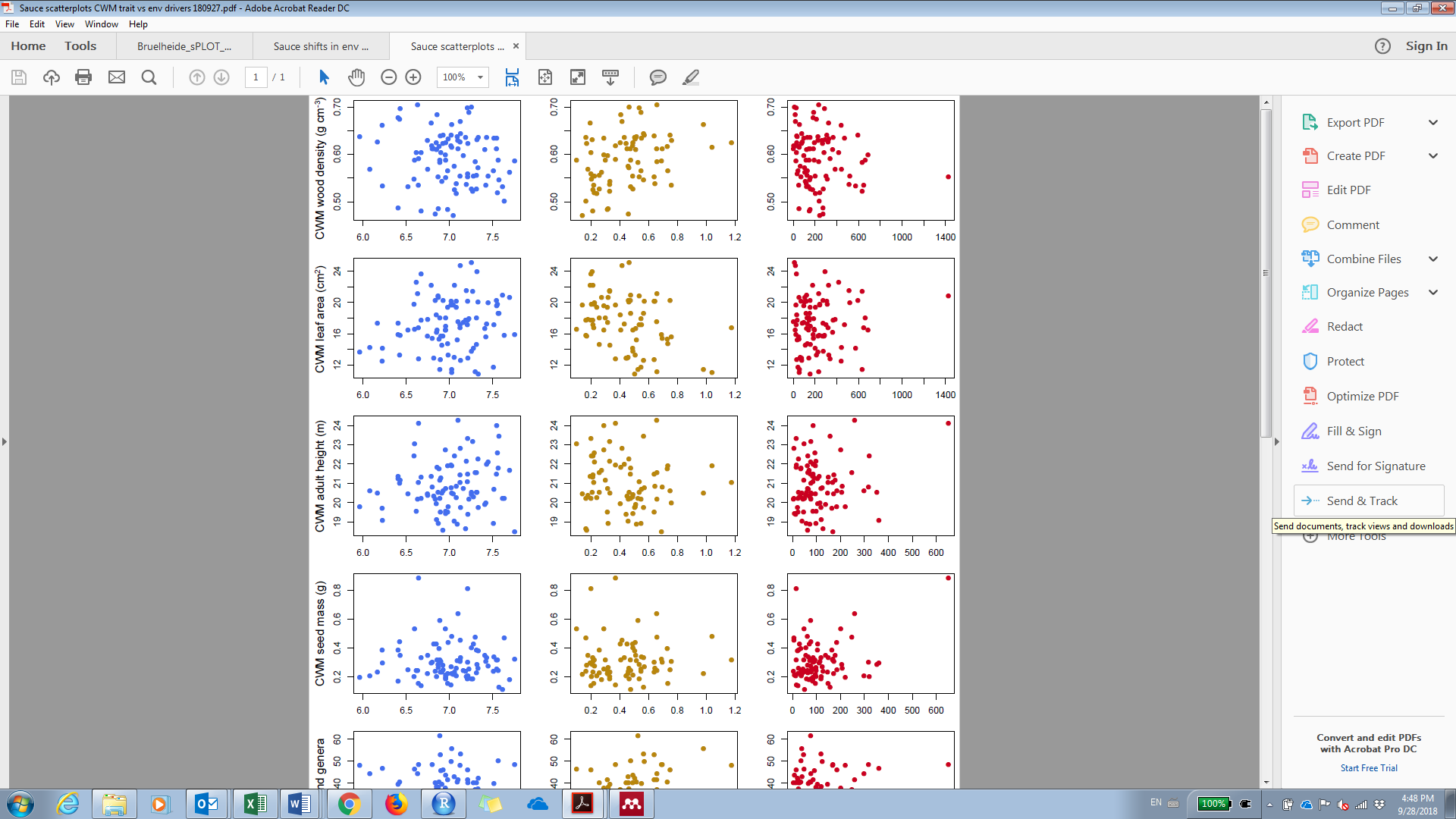  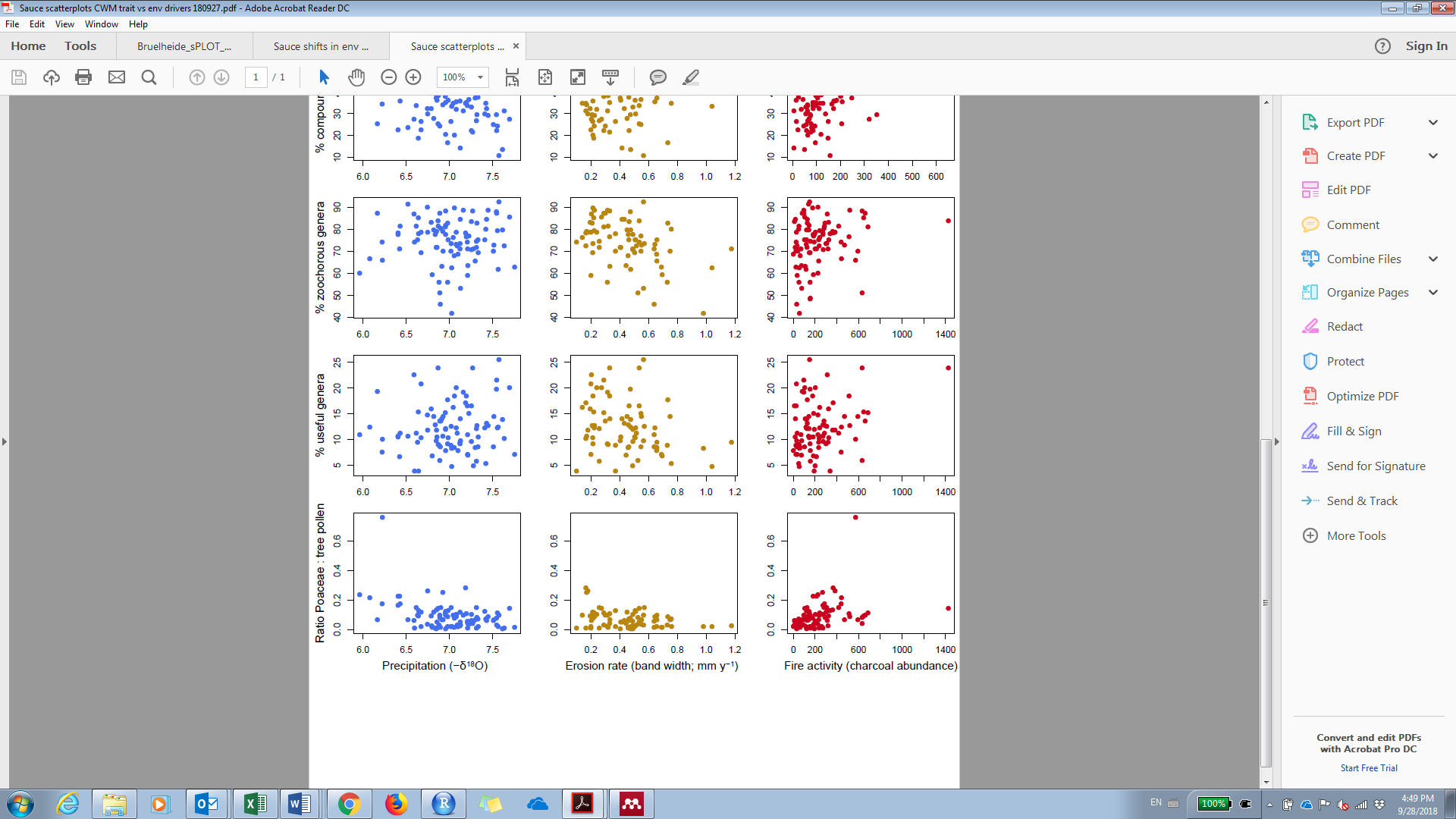 |
| --- |

**Appendix S12:** Pearson correlation coefficients between environmental variables: precipitation, erosion rate, and fire activity (measured using the previous event and the maximum event in the preceding time period, see Methods).

|  | Erosion rate | Fire activity (previous) | Fire activity (maximum) |
| --- | --- | --- | --- |
| Precipitation | 0.30 | -0.30 | -0.22 |
| Erosion rate |  | -0.03 | -0.17 |
| Fire activity (previous) |  |  | 0.31 |

**References:**

van Breukelen, M.R., Vonhof, H.B., Hellstrom, J.C., Wester, W.C.G. & Kroon, D. (2008). Fossil dripwater in stalagmites reveals Holocene temperature and rainfall variation in Amazonia. *Earth Planet. Sci. Lett.*, 275, 54–60.

Pearse, W.D., Cadotte, M.W., Cavender-Bares, J., Ives, A.R., Tucker, C.M., Walker, S.C., *et al.* (2015). Pez: Phylogenetics for the environmental sciences. *Bioinformatics*, 31, 2888–2890.

van der Sande, M.T., Gosling, W., Correa-Metrio, A., Prado-Junior, J., Poorter, L., Oliveira, R.S., *et al.* (n.d.). A 7000-year history of changing plant trait composition in an Amazonian landscape; the role of humans and climate. *Rev.*

Zanne, A.E., Tank, D.C., Cornwell, W.K., Eastman, J.M., Smith, S.A., FitzJohn, R.G., *et al.* (2014). Three keys to the radiation of angiosperms into freezing environments. *Nature*, 506, 89–92.

Zeileis, A., Leisch, F., Hornik, K., Kleiber, C., Hansen, B. & Merkle, E.C. (2015). *Package “strucchange”: testing, monitoring, and dating structural changes*.

Zelený, D. & Schaffers, A.P. (2012). Too good to be true: Pitfalls of using mean Ellenberg indicator values in vegetation analyses. *J. Veg. Sci.*, 23, 419–431.
